# Supplementary material for: Identification of photoperiod-induced specific miRNAs in the adrenal glands of Sunite sheep (Ovis aries)
Source: Front Vet Sci. 2022 Jul 22;9:888207. doi: 10.3389/fvets.2022.888207 (PMC9354845; doi:10.3389/fvets.2022.888207)
Supplement: Supplementary file 1 [file Data_Sheet_1.ZIP › Supplementary file/miRDeep2 file.zip!/miRDeep2 file/Know_Structure/SP42A2/oar-mir-665.pdf]

miRBase precursor : oar-mir-665
Total read count : 106
oar-miR-665-5p read count : 2
oar-miR-665-3p read count : 100
remaining reads : 4

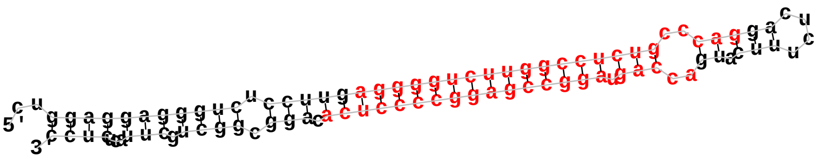

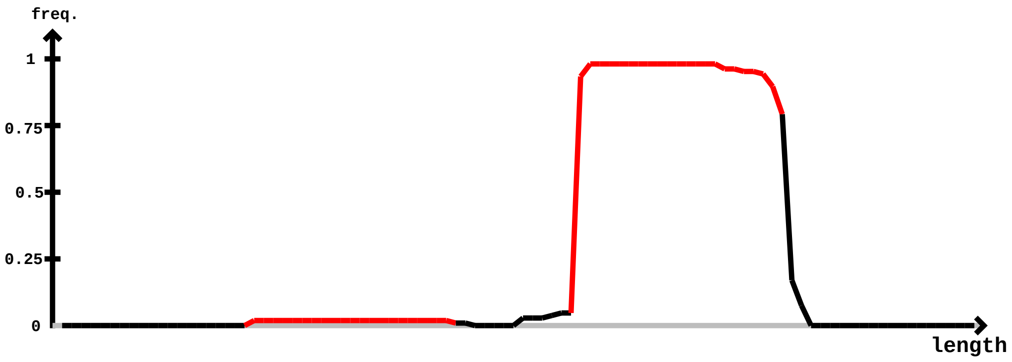


```
                                                        oar-miR-665-3p
                    oar-miR-665-5p
5'- cuggaggagggucuccuugaggggucuuggccucugcccaggacucuuucaugaccaguaggccgaggccccucacaggcggcugcuuacucucc   -3'    exp
    ..((((((((((.((((((((((((((((((((((..(((((....))).))..))).))))))))))))))))).))).)))).))).))))..   reads       mm          sample
    ...................aggggucuuggccucugccca.......................................................   1           0           seq
    ...................aggggucuuggccucugcccagg.....................................................   1           0           seq
    ...............................................uucaugaccaguaggccgagg...........................   2           0           seq
    ...............................................uucaugaccaguaggccgaggcc.........................   1           0           seq
    ..................................................augaccaguaggccgaggccccuc.....................   1           0           seq
    ...................................................ugaccaguaggccgaggccccuc.....................   1           0           seq
    .....................................................accaguaggccgaggcccc.......................   1           0           seq
    .....................................................accaguaggccgaggccccu......................   5           0           seq
    .....................................................accaguaggccgaggccccuc.....................   9           0           seq
    .....................................................accaguaggccgaggccccuca....................   64          0           seq
    .....................................................accaguaggccgaggccccucac...................   9           0           seq
    .....................................................accaguaggccgaggccccucaca..................   6           0           seq
    ......................................................ccaguaggccgaggccccuca....................   2           0           seq
    ......................................................ccaguaggccgaggccccucac...................   1           0           seq
    ......................................................ccaguaggccgaggccccucaca..................   2           0           seq
```
